# *N*-[(Piperidin-1-yl)carbothioyl]benz­amide

**DOI:** 10.1107/S160053681004170X

**Published:** 2010-10-23

**Authors:** Aisha A. Al-abbasi, Mohd Ambar Yarmo, Mohammad B. Kassim

**Affiliations:** a School of Chemical Sciences and Food Technology, Faculty of Science and Technology, Universiti Kebangsaan Malaysia, UKM 43600 Bangi Selangor, Malaysia

## Abstract

In the title compound, C_13_H_16_N_2_OS, the piperidine ring exhibit a classical chair conformation. In the crystal, the mol­ecules are linked by N—H⋯O hydrogen bonds, forming zigzag chains running parallel to the *c* axis.

## Related literature

For complexes with the title compound as a ligand, see: Mohamadou *et al.* (1994 ▶); Salyn *et al.* (1977 ▶); Röbisch *et al.* (1982 ▶).
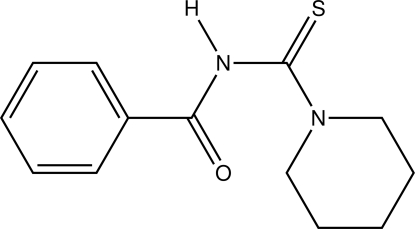


## Experimental

### 

#### Crystal data

C_13_H_16_N_2_OS
*M* *_r_* = 248.34
Monoclinic, 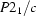

*a* = 10.913 (3) Å
*b* = 14.297 (4) Å
*c* = 8.323 (2) Å
β = 102.212 (6)°
*V* = 1269.2 (6) Å^3^
*Z* = 4
Mo *K*α radiation
μ = 0.24 mm^−1^
*T* = 298 K
0.50 × 0.41 × 0.38 mm

#### Data collection

Bruker SMART APEX CCD area-detector diffractometer
Absorption correction: multi-scan (*SADABS*; Bruker, 2000 ▶) *T*_min_ = 0.889, *T*_max_ = 0.915
7091 measured reflections
2221 independent reflections
1727 reflections with *I* > 2σ(*I*)
*R*_int_ = 0.031

#### Refinement

*R*[*F* ^2^ > 2σ(*F* ^2^)] = 0.047
*wR*(*F* ^2^) = 0.126
*S* = 1.08
2221 reflections
154 parameters
H-atom parameters constrained
Δρ_max_ = 0.29 e Å^−3^
Δρ_min_ = −0.27 e Å^−3^

### 

Data collection: *SMART* (Bruker, 2000 ▶); cell refinement: *SAINT* (Bruker, 2000 ▶); data reduction: *SAINT*; program(s) used to solve structure: *SHELXS97* (Sheldrick, 2008 ▶); program(s) used to refine structure: *SHELXL97* (Sheldrick, 2008 ▶); molecular graphics: *XP* (Sheldrick, 2008 ▶); software used to prepare material for publication: *SHELXTL* (Sheldrick, 2008 ▶), *PARST* (Nardelli, 1995 ▶) and *PLATON* (Spek, 2009 ▶).

## Supplementary Material

Crystal structure: contains datablocks I, global. DOI: 10.1107/S160053681004170X/bt5376sup1.cif

Structure factors: contains datablocks I. DOI: 10.1107/S160053681004170X/bt5376Isup2.hkl

Additional supplementary materials:  crystallographic information; 3D view; checkCIF report

## Figures and Tables

**Table 1:** Hydrogen-bond geometry (Å, °)

*D*—H⋯*A*	*D*—H	H⋯*A*	*D*⋯*A*	*D*—H⋯*A*
N1—H1*A*⋯O1^i^	0.86	2.18	2.949 (2)	149

Symmetry code: (i) 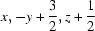.

**Fig. 1.**
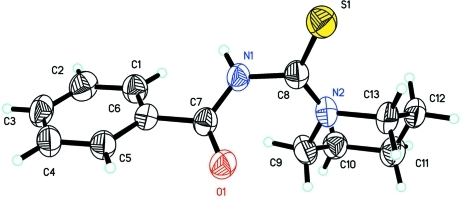
The molecular structure of N-(piperidine-1-carbamothioyl)benzamide, with displacement ellipsoids are drawn at the 50% probability level.

**Fig. 2.**
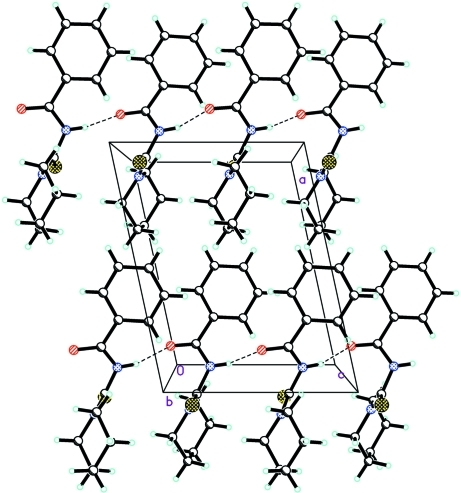
Crystal packing of the title compound viewed down the c-axis. Hydrogen bonds are drawn as dashed lines.

## supplementary crystallographic information

### Comment

*N,N-*dialkyl-*N'*-benzoyl thioureas are known to form stable complexes with lage number of transition metals. The title compound has been used extensivelly as ligand to form stable complexes with Cu, Ni, Co, Pt, Pd, Hg, Ru, Os, Rh and Ir (Mohamadou *et al.*, 1994; Salyn *et al.*, 1977; Röbisch *et al.*, 1982). The six-membered piperidine ring has a classical chair conformation.

In the crystal, the molecules are linked by N—H···O hydrogen bonds forming zigzag chains running parallel to the crystallographic c-axis.

### Experimental

A solution of benzoyl chloride (10 mmol) in acetone was added slowly to a equimolar solution of ammonium thiocyanate in acetone. The reaction mixture was stirred at room temperature before adding piperidine (10 mmol) slowly and left stirring at room temperature for 4 h. The mixture was poured on to a water-ice mixture and then filtered. The pure product was recrystallized to give colourless crystals (70% yield).

### Refinement

H atom positions were  calculated and they were refined using a  riding model with *U*_iso_=1.2*U*_eq_(C,N) and with C_aromatic_-H = 0.93 Å or C-H = 0.97 Å, and N-H = 0.86 Å.

### Crystal data

**Table:**

C_13_H_16_N_2_OS	*F*(000) = 528
*M**_r_* = 248.34	*D*_x_ = 1.300 Mg m^−^^3^
Monoclinic, *P*2_1_/*c*	Mo *K*α radiation, λ = 0.71073 Å
Hall symbol: -P 2ybc	Cell parameters from 1679 reflections
*a* = 10.913 (3) Å	θ = 1.9–25.0°
*b* = 14.297 (4) Å	µ = 0.24 mm^−^^1^
*c* = 8.323 (2) Å	*T* = 298 K
β = 102.212 (6)°	Needle, colourless
*V* = 1269.2 (6) Å^3^	0.50 × 0.41 × 0.38 mm
*Z* = 4	

### Data collection

**Table:**

Bruker SMART APEX CCD area-detector diffractometer	2221 independent reflections
Radiation source: fine-focus sealed tube	1727 reflections with *I* > 2σ(*I*)
graphite	*R*_int_ = 0.031
ω scan	θ_max_ = 25.0°, θ_min_ = 1.9°
Absorption correction: multi-scan (*SADABS*; Bruker, 2000)	*h* = −12→8
*T*_min_ = 0.889, *T*_max_ = 0.915	*k* = −16→17
7091 measured reflections	*l* = −9→9

### Refinement

**Table:**

Refinement on *F*^2^	Primary atom site location: structure-invariant direct methods
Least-squares matrix: full	Secondary atom site location: difference Fourier map
*R*[*F*^2^ > 2σ(*F*^2^)] = 0.047	Hydrogen site location: inferred from neighbouring sites
*wR*(*F*^2^) = 0.126	H-atom parameters constrained
*S* = 1.08	*w* = 1/[σ^2^(*F*_o_^2^) + (0.0702*P*)^2^ + 0.1087*P*] where *P* = (*F*_o_^2^ + 2*F*_c_^2^)/3
2221 reflections	(Δ/σ)_max_ < 0.001
154 parameters	Δρ_max_ = 0.29 e Å^−^^3^
0 restraints	Δρ_min_ = −0.27 e Å^−^^3^

### Special details

**Table:**

Geometry. All e.s.d.'s (except the e.s.d. in the dihedral angle between two l.s. planes) are estimated using the full covariance matrix. The cell e.s.d.'s are taken into account individually in the estimation of e.s.d.'s in distances, angles and torsion angles; correlations between e.s.d.'s in cell parameters are only used when they are defined by crystal symmetry. An approximate (isotropic) treatment of cell e.s.d.'s is used for estimating e.s.d.'s involving l.s. planes.
Refinement. Refinement of *F*^2^ against ALL reflections. The weighted *R*-factor *wR* and goodness of fit *S* are based on *F*^2^, conventional *R*-factors *R* are based on *F*, with *F* set to zero for negative *F*^2^. The threshold expression of *F*^2^ > σ(*F*^2^) is used only for calculating *R*-factors(gt) *etc*. and is not relevant to the choice of reflections for refinement. *R*-factors based on *F*^2^ are statistically about twice as large as those based on *F*, and *R*- factors based on ALL data will be even larger.

### Fractional atomic coordinates and isotropic or equivalent isotropic displacement parameters (Å^2^)

**Table:**

	*x*	*y*	*z*	*U*_iso_*/*U*_eq_	
S1	0.93006 (6)	0.86988 (4)	0.12334 (8)	0.0562 (2)	
O1	1.15395 (14)	0.65828 (11)	0.05387 (17)	0.0500 (4)	
N1	1.07366 (15)	0.72851 (13)	0.2538 (2)	0.0419 (4)	
H1A	1.0864	0.7427	0.3564	0.050*	
N2	0.88426 (17)	0.68658 (13)	0.0845 (2)	0.0444 (5)	
C1	1.2732 (2)	0.63954 (15)	0.4875 (3)	0.0472 (6)	
H1B	1.1968	0.6424	0.5201	0.057*	
C2	1.3821 (3)	0.61791 (16)	0.6010 (3)	0.0546 (6)	
H2A	1.3790	0.6072	0.7102	0.066*	
C3	1.4937 (2)	0.61231 (16)	0.5526 (3)	0.0560 (7)	
H3A	1.5665	0.5981	0.6294	0.067*	
C4	1.4996 (2)	0.62748 (16)	0.3912 (3)	0.0526 (6)	
H4A	1.5760	0.6230	0.3590	0.063*	
C5	1.3923 (2)	0.64924 (15)	0.2774 (3)	0.0448 (5)	
H5A	1.3961	0.6589	0.1681	0.054*	
C6	1.27884 (19)	0.65682 (14)	0.3256 (2)	0.0385 (5)	
C7	1.1646 (2)	0.68059 (14)	0.1983 (2)	0.0393 (5)	
C8	0.95923 (19)	0.75579 (16)	0.1496 (2)	0.0404 (5)	
C9	0.9072 (2)	0.58599 (16)	0.1188 (3)	0.0521 (6)	
H9A	0.9804	0.5785	0.2071	0.062*	
H9B	0.9243	0.5554	0.0218	0.062*	
C10	0.7954 (2)	0.53997 (16)	0.1670 (3)	0.0502 (6)	
H10A	0.8099	0.4731	0.1782	0.060*	
H10B	0.7864	0.5640	0.2729	0.060*	
C11	0.6756 (2)	0.55748 (17)	0.0417 (3)	0.0576 (7)	
H11A	0.6051	0.5319	0.0813	0.069*	
H11B	0.6797	0.5264	−0.0605	0.069*	
C12	0.6569 (2)	0.66148 (17)	0.0119 (3)	0.0511 (6)	
H12A	0.5826	0.6717	−0.0736	0.061*	
H12B	0.6438	0.6914	0.1115	0.061*	
C13	0.7685 (2)	0.70500 (17)	−0.0388 (3)	0.0491 (6)	
H13A	0.7767	0.6796	−0.1441	0.059*	
H13B	0.7559	0.7720	−0.0514	0.059*	

### Atomic displacement parameters (Å^2^)

**Table:**

	*U*^11^	*U*^22^	*U*^33^	*U*^12^	*U*^13^	*U*^23^
S1	0.0585 (4)	0.0490 (4)	0.0571 (4)	0.0034 (3)	0.0029 (3)	0.0042 (3)
O1	0.0432 (9)	0.0702 (11)	0.0352 (9)	0.0035 (8)	0.0048 (7)	−0.0055 (7)
N1	0.0350 (10)	0.0584 (11)	0.0305 (9)	0.0052 (9)	0.0028 (7)	−0.0008 (7)
N2	0.0314 (10)	0.0495 (11)	0.0489 (10)	0.0030 (9)	0.0009 (8)	0.0062 (8)
C1	0.0428 (13)	0.0551 (14)	0.0431 (13)	0.0013 (11)	0.0078 (10)	0.0043 (10)
C2	0.0624 (16)	0.0561 (15)	0.0399 (13)	0.0032 (13)	−0.0013 (11)	0.0079 (10)
C3	0.0446 (14)	0.0504 (14)	0.0619 (16)	0.0031 (11)	−0.0136 (12)	0.0039 (11)
C4	0.0352 (13)	0.0545 (15)	0.0653 (16)	0.0000 (11)	0.0044 (11)	−0.0026 (11)
C5	0.0383 (12)	0.0496 (13)	0.0456 (12)	−0.0010 (10)	0.0065 (10)	−0.0002 (10)
C6	0.0341 (12)	0.0411 (11)	0.0387 (11)	−0.0015 (9)	0.0042 (9)	0.0004 (9)
C7	0.0342 (12)	0.0475 (12)	0.0359 (11)	−0.0035 (10)	0.0065 (9)	0.0032 (9)
C8	0.0351 (11)	0.0556 (13)	0.0313 (10)	0.0023 (10)	0.0084 (9)	0.0030 (9)
C9	0.0374 (13)	0.0471 (13)	0.0689 (16)	0.0065 (11)	0.0051 (11)	0.0026 (11)
C10	0.0447 (14)	0.0438 (12)	0.0586 (13)	−0.0018 (11)	0.0033 (11)	0.0000 (10)
C11	0.0454 (14)	0.0603 (15)	0.0625 (15)	−0.0081 (12)	0.0012 (12)	−0.0025 (12)
C12	0.0338 (12)	0.0640 (15)	0.0505 (13)	0.0001 (11)	−0.0027 (10)	−0.0025 (11)
C13	0.0396 (13)	0.0599 (14)	0.0420 (12)	0.0039 (11)	−0.0041 (10)	0.0062 (10)

### Geometric parameters (Å, °)

**Table:**

S1—C8	1.667 (2)	C5—C6	1.385 (3)
O1—C7	1.225 (2)	C5—H5A	0.9300
N1—C7	1.364 (3)	C6—C7	1.495 (3)
N1—C8	1.416 (3)	C9—C10	1.514 (3)
N1—H1A	0.8600	C9—H9A	0.9700
N2—C8	1.325 (3)	C9—H9B	0.9700
N2—C13	1.473 (3)	C10—C11	1.511 (3)
N2—C9	1.477 (3)	C10—H10A	0.9700
C1—C6	1.385 (3)	C10—H10B	0.9700
C1—C2	1.388 (3)	C11—C12	1.514 (3)
C1—H1B	0.9300	C11—H11A	0.9700
C2—C3	1.364 (4)	C11—H11B	0.9700
C2—H2A	0.9300	C12—C13	1.506 (3)
C3—C4	1.376 (3)	C12—H12A	0.9700
C3—H3A	0.9300	C12—H12B	0.9700
C4—C5	1.377 (3)	C13—H13A	0.9700
C4—H4A	0.9300	C13—H13B	0.9700
			
C7—N1—C8	122.74 (17)	N2—C9—C10	111.15 (18)
C7—N1—H1A	118.6	N2—C9—H9A	109.4
C8—N1—H1A	118.6	C10—C9—H9A	109.4
C8—N2—C13	121.05 (18)	N2—C9—H9B	109.4
C8—N2—C9	125.68 (18)	C10—C9—H9B	109.4
C13—N2—C9	113.22 (18)	H9A—C9—H9B	108.0
C6—C1—C2	119.6 (2)	C11—C10—C9	111.9 (2)
C6—C1—H1B	120.2	C11—C10—H10A	109.2
C2—C1—H1B	120.2	C9—C10—H10A	109.2
C3—C2—C1	120.2 (2)	C11—C10—H10B	109.2
C3—C2—H2A	119.9	C9—C10—H10B	109.2
C1—C2—H2A	119.9	H10A—C10—H10B	107.9
C2—C3—C4	120.5 (2)	C10—C11—C12	110.07 (19)
C2—C3—H3A	119.7	C10—C11—H11A	109.6
C4—C3—H3A	119.7	C12—C11—H11A	109.6
C3—C4—C5	119.9 (2)	C10—C11—H11B	109.6
C3—C4—H4A	120.0	C12—C11—H11B	109.6
C5—C4—H4A	120.0	H11A—C11—H11B	108.2
C4—C5—C6	120.1 (2)	C13—C12—C11	111.2 (2)
C4—C5—H5A	120.0	C13—C12—H12A	109.4
C6—C5—H5A	120.0	C11—C12—H12A	109.4
C1—C6—C5	119.7 (2)	C13—C12—H12B	109.4
C1—C6—C7	121.89 (19)	C11—C12—H12B	109.4
C5—C6—C7	118.43 (18)	H12A—C12—H12B	108.0
O1—C7—N1	122.59 (19)	N2—C13—C12	110.88 (18)
O1—C7—C6	121.86 (19)	N2—C13—H13A	109.5
N1—C7—C6	115.56 (17)	C12—C13—H13A	109.5
N2—C8—N1	115.66 (19)	N2—C13—H13B	109.5
N2—C8—S1	126.36 (16)	C12—C13—H13B	109.5
N1—C8—S1	117.96 (16)	H13A—C13—H13B	108.1
			
C6—C1—C2—C3	−1.0 (3)	C13—N2—C8—N1	−173.80 (17)
C1—C2—C3—C4	−0.3 (4)	C9—N2—C8—N1	3.5 (3)
C2—C3—C4—C5	0.5 (3)	C13—N2—C8—S1	7.6 (3)
C3—C4—C5—C6	0.7 (3)	C9—N2—C8—S1	−175.12 (16)
C2—C1—C6—C5	2.2 (3)	C7—N1—C8—N2	65.2 (3)
C2—C1—C6—C7	−179.9 (2)	C7—N1—C8—S1	−116.00 (19)
C4—C5—C6—C1	−2.0 (3)	C8—N2—C9—C10	128.2 (2)
C4—C5—C6—C7	179.98 (19)	C13—N2—C9—C10	−54.3 (2)
C8—N1—C7—O1	0.5 (3)	N2—C9—C10—C11	53.4 (3)
C8—N1—C7—C6	−179.52 (18)	C9—C10—C11—C12	−54.2 (3)
C1—C6—C7—O1	−148.6 (2)	C10—C11—C12—C13	55.5 (3)
C5—C6—C7—O1	29.4 (3)	C8—N2—C13—C12	−126.5 (2)
C1—C6—C7—N1	31.4 (3)	C9—N2—C13—C12	55.8 (2)
C5—C6—C7—N1	−150.61 (19)	C11—C12—C13—N2	−56.1 (3)

### Hydrogen-bond geometry (Å, °)

**Table:**

*D*—H···*A*	*D*—H	H···*A*	*D*···*A*	*D*—H···*A*
N1—H1A···O1^i^	0.86	2.18	2.949 (2)	149

Symmetry codes: (i) *x*, −*y*+3/2, *z*+1/2.
